# Evaluating the efficacy and impact of neutropenic diet in pediatric hematology patients: a longitudinal cohort study on adherence, clinical outcomes, and socioeconomic factors

**DOI:** 10.3389/fnut.2025.1533734

**Published:** 2025-03-17

**Authors:** Amitabh Singh, Neetu Kushwaha, Raja Srishwan, Shamsuz Zaman, Noreen Grace George, Raj Kamal, Sandeep Kumar Swain, Manpreet Kaur, Fouzia Siraj, Saurabh Sharma, Baseer Noor, Prashant Prabhakar, Bhavika Rishi, Aroonima Misra

**Affiliations:** ^1^Vardhman Mahavir Medical College and Safdarjung Hospital, New Delhi, India; ^2^ICMR-National Institute of Child Health and Development Research, New Delhi, India; ^3^ICMR-National Institute of Nutrition, Hyderabad, India; ^4^ICMR-National Institute of Cancer Prevention and Research, Noida, India; ^5^ICMR-Centre for Cancer Pathology, New Delhi, India; ^6^Indian Council of Medical Research (ICMR), New Delhi, India

**Keywords:** neutropenic diet, leukemia, febrile neutropenia, pediatric patients, adherence, socio-economic factors

## Abstract

**Background and aim:**

A neutropenic diet aims to reduce hospitalizations from febrile neutropenia and sepsis in pediatric hematology patients during chemotherapy. This study aimed to evaluate its effectiveness in improving mortality, morbidity, and overall outcomes while considering limitations, adherence rates, and its impact on hospital admissions and culture positivity.

**Method:**

A prospective 18-month observational study was conducted on pediatric hematology patients in a pediatric department at a tertiary care center. Using a baseline questionnaire at the introduction of a neutropenic diet, the study assessed the clinical history, diagnosis, clinicopathological parameters, dietary recommendations, and socio-demographic data of the patients. Patients were followed up for up to 1 year to evaluate diet adherence, outcomes, mortality, and morbidity, as indicated by hospital admissions for febrile neutropenia.

**Results:**

An analysis involving 100 patients was conducted to assess adherence to a neutropenic diet and its ramifications on clinical outcomes over a period of 18 months. Initial follow-up data were accessible for 83 patients, revealing an adherence rate of 66%, which subsequently declined to 57% following a 6-month interval. Patients were categorized as compliant or non-compliant, but no correlation was found between adherence and febrile admissions, sepsis, hospitalizations, or mortality. Among compliant patients, 62% showed sepsis signs, though only 19% had positive blood cultures in the whole study group. Non-adherence was linked to demographic factors such as large family size, financial constraints, and limited resources. The neutropenic diet showed minimal impact on morbidity and mortality.

**Conclusion:**

Our study does not support the strict adherence to the neutropenic diet, as there is no evidence of reduced infections and the dietary adherence also imposes an undue financial burden on patients. Instead, focusing on the safe acquisition of food, food processing, and proper hand cleanliness will probably provide superior protection against infection.

## Introduction

Childhood cancers have high mortality & morbidity owing to factors that are genetic, idiopathic, disease pathogenesis related and those due to to chemotherapy related toxicity (1, 2). The prevalence of morbidity and mortality in hemato-proliferative disorders is often attributed to febrile neutropenia which is the most common sequel of chemotherapy-induced toxicity (3). Therefore, numerous studies have been conducted to improve the various factors that contribute to morbidity and mortality. A neutropenic diet (also called a low microbial diet) is a clinically recommended special diet on permissible food items and is believed to reduce the risk of infection in cancer patients and patients with compromised immune systems (4). This diet eliminates raw produce, soft cheeses, fast food, and other potentially contaminated foods, thus reducing the risk of infections. The clinical practice of a neutropenic diet for cancer patients remains controversial, despite its lack of scientific evidence, as it is still followed in the majority of cancer centers for preventing neutropenia-related infections during chemotherapy (2, 5–7). There is a paucity of empirical evidence regarding the efficacy of this dietary regimen in hematological disorders prevalent in the Indian population, with limited studies assessing its role in preventing sepsis, chemotherapy-induced febrile neutropenia, and its impact on long-term mortality and morbidity in these patients (4).

The efficacy of the neutropenic diet in mitigating the incidence of infections among patients undergoing chemotherapy remains debated in the Indian population. Theoretically, in a setting such as tropical countries like India, which have a high burden of bacterial and infectious agents due to elevated humidity and environmental conditions, a neutropenic diet could be anticipated to significantly reduce morbidity, even if not mortality. Research has identified Gram-negative bacteria, such as *Pseudomonas aeruginosa*, *Escherichia coli*, *Klebsiella*, and *Proteus*, in various foods, especially salads, fresh vegetables, and cold meats. In addition, *Aspergillus*, a potentially deadly fungus for those with extended neutropenia, has been identified in food, water, and ice (6). However, the recent oncology practice worldwide has seen a shift from strict dietary adherence to relaxation of such adherence considering the studies on these diets and the overall marginal resultant improvement in morbidity (8, 9).

There are limited studies in India to validate or refute this hypothesis. Therefore, we conducted a study to determine the impact of a neutropenic diet on a cohort of patients undergoing chemotherapy for acute lymphoblastic leukemia (ALL), acute myeloid leukemia (AML), and other hematology disorders, and we analyzed the correlation between adherence to the neutropenic diet and morbidity outcomes. Thus, the goal of this mixed-methods study was to assess the neutropenic diet intervention in pediatric cancer patients with their clinical outcomes and admission related to febrile neutropenia and clinicopathological parameters, in order to support clinical and nutritional strategies that may improve the chemotherapy patients’ quality of life (2). Our study also aimed to assess the feasibility of implementing a neutropenic diet, particularly in hematological malignancies, where the effectiveness of dietary intervention is established, but socioeconomic barriers often hinder adherence.

## Methods

### Study design and patients

The study was conducted at the Indian Council of Medical Research (ICMR)-National Institute of Child Health & Development Research (NICHDR), New Delhi. Pediatric patients under the age of 13 years who were admitted to the pediatric ward at Vardhman Mahavir Medical College (VMMC) and Safdarjung Hospital for chemotherapy treatment of hematological malignancies were included in the study. The child’s age, medical diagnosis, and therapy stage were documented with records for weight, height, hemoglobin level, platelet count, total leukocyte count, absolute neutrophil count, and temperature. Demographic data included age, sex, birth date, admission date, patient’s and mother’s physiological status, and examination date. In addition, parental income, employment status, and educational background of parents were collected. The baseline data included medical history (diagnosis and its date, surgeries, etc.), neutropenic infection history, and prior dietary adherence.

Samples and qualitative data were collected from the first day of chemotherapy. The blood samples were taken for various tests including hemoglobin (Hb), absolute neutrophil count, platelet count, total leucocyte count (TLC), liver function test (LFT), kidney function test (KFT), and blood culture. Patient data were recorded at the time of admission as well as at the start of chemotherapy and the initiation of a neutropenic diet and then subsequently at 6 months and/or upon hospital admission due to any disease-related morbidity, whichever transpired first. The second follow-up assessment was conducted 1 year subsequent to the initiation of therapy and the neutropenic diet.

### Diet intervention at the start of chemotherapy

The investigator explained the details of the diet to the patients and their parents through the guidelines, answering any queries. The details of the diet to be followed and also the dietary intervention and its precautions are described in Supplementary Table 9. On the first day of the chemotherapy cycle, patients were instructed to start their diet, until the chemotherapy ended. Patients on a neutropenic diet were instructed to avoid leftover foods, fresh fruits, and raw vegetables. The baseline data of all 100 patients were collected at the time of initiation of the neutropenic diet and admission at Vardhman Mahavir Medical College, Safdarjung Hospital. The first follow-up of all the patients was done after 6 weeks, and the second follow-up was done after 3 months from the date of initiation of the neutropenic diet wherever feasible. In addition, in the case of hospital admission, data were collected for the follow-up questionnaire to inquire about the adherence to neutropenic diet and the correlated clinicopathological results for sepsis and related admission.

The principal investigator evaluated hospital admission history, physical examination, and laboratory results to determine the diagnosis, including chemotherapy, and non-neutropenic and neutropenic infections. The lead investigator obtained a complete history from parents of hospitalized patients with neutropenic infections and documented all tests and results including systemic laboratory examinations and culture reports from blood. Patients’ blood counts were monitored throughout the treatment until neutrophil recovery, indicated by an absolute neutrophil count (ANC), with fever detected and hospitalization if found.

The following data were collected at the time of baseline and follow-up: adherence to diet, reasons for non-compliance, and details about the diet such as intake of leftover foods, packaged foods, vegetarian or non-vegetarian foods, fresh fruits, and raw vegetables. Then, similar data were recorded at the time of the first follow-up and second follow-up. The questionnaire (see Supplementary Table 5) was based on the guidelines (see Supplementary Table 9) of the neutropenic diet, and the data were collected telephonically or at the time of admission or during OPD visits of all the patients. The 24-h diet recall approach was used to assess diet tenacity, with weekly interviews with parents to assess their child’s adherence to the recommended food intake.

Neutropenic infection was chosen as the primary outcome, and it was defined as febrile neutropenia, requiring hospital admission, broad-spectrum antibiotic treatment, and an oral temperature of 38°C or above. Secondary outcomes included documented infections such as pneumonia or positive blood, urine, stool, or sputum cultures. The correlation with adherence to the neutropenic diet was determined for these patients. In addition, the following data were collected from the record files of the patients: ANC count, Hb level, blood culture report, incidence of hospital admission due to febrile neutropenia, and other investigations, as mentioned in Supplementary Tables 1, 2.

### Statistical analysis for calculation of the risk ratio between the compliant and non-compliant groups

The statistical analysis of the study was conducted using Jamovi-open statistics software (Version: 2.3.28, Solid). A *p*-value less than 0.05 was the threshold for statistical significance. The study was conducted using the logarithmic risk ratio as the measure of final outcomes. The data were fitted with a random-effects statistical model. We evaluated the level of heterogeneity (tau^2^) using the constrained maximum-likelihood estimator. In addition to the tau^2^ estimate, the Q-test for heterogeneity and the I^2^ statistic were also provided. If any degree of heterogeneity was observed (i.e., tau^2^ > 0 irrespective of the Q-test findings), a prediction interval for the actual effects was also given. The utilization of studentized residuals and Cook’s distance aided in the examination of potential outliers and/or influential studies within the model’s context. Studies exhibiting a studentized residual in excess of the 100 × (1–0.05/ (2 × k))th percentile of a regular normal distribution are regarded as possible outliers. This is determined by applying a Bonferroni correction with a two-sided alpha of 0.05 to the k studies included in the meta-analysis (10, 11).

## Results

### General characteristics of the study population

Out of 100 patients recruited for the study, the second follow-up data were available for 74 patients and the first follow-up data were available for 26 patients recruited at a later part of the study. Our tertiary care setup included a team of senior and junior hemato-oncologists, junior and senior doctors, nurse staff, and supporting staff. Supplementary Table 1 provides the characteristics of the pediatric children at our hospital. Among the 100 pediatric hematology patients, we observed a slight male predominance in hemato-proliferative disorders, with a higher proportion of males (61%) (see Supplementary Tables 1, 3). The reasons for this predominance may include tertiary referral bias and a greater likelihood of a male child receiving medical attention because of socioeconomic factors in India (12). The median age of patients was 5.32 years. The majority of patients were in the age group of 0–5 years (48%) followed by 5–10 years old (31%). The most common type of leukemia in this population was B-ALL (55%) followed by T-ALL (13%) (Supplementary Tables 1, 3). The results of this demography distribution were similar to those reported in previous studies (7, 9, 13, 14). The study also found that literacy could also be a potential confounding factor for the implementation of a neutropenic diet in daily practice, which plays a major role in understanding the importance of a neutropenic diet in leukemia. The compliance was better in the group where both parents were literate than in the group where either or none was literate (with 38% being primary passed and 24% illiterate) (15, 16). The majority of children treated were underweight. Supplementary Table 4 provides the details of the social demographics of the study population.

### Presenting symptoms and clinicopathological data of patients

Supplementary Table 2 provides details of the clinical presentation of the study population at baseline. The majority of hematology patients were diagnosed with hepatosplenomegaly (63%), hepatomegaly (16%), splenomegaly (2%), hydronephrosis (1%), and lymphadenopathy (64%). Out of 100 patients, almost all of them experienced fever, the majority (75%) had pallor, followed by abdominal pain, easy bruises, bleeding, and vomiting during admission.

### Neutropenic infection

In our study, 91% of patients often developed neutropenic infections and vomiting, believed to be the side effects of chemotherapy, during the entire study period and follow-ups (Supplementary Figure 3). Patients reported experiencing fever (97%), vomiting (25%), and diarrhea (38%) at the time of admission due to febrile neutropenia. We found that 19% of patients showed positive blood culture and 91% of patients had sepsis (Supplementary Table 2). All of these were unrelated to neutropenic diet adherence and were randomly distributed in both adherent and non-adherent groups. One patient had oral candidiasis with *E. coli* sepsis and MRSA sepsis. Another patient had *Acinetobacter* sepsis. One patient had febrile neutropenia with *Klebsiella* sepsis. The majority of the patients on a neutropenic diet showed normal blood cultures, but neutropenic infections also occurred in some individuals (17, 18). There was a lack of laboratory evidence supporting the diagnosis of sepsis, as the majority of cultures obtained from patients admitted with febrile neutropenia yielded negative results. This phenomenon is corroborated by additional research indicating the occurrence of culture-negative sepsis among individuals undergoing chemotherapy (15, 17, 18).

This study found no difference in mortality, indicating that diet does not play a significant role in reducing infection and mortality in oncology treatment patients (Supplementary Figure 4), when comparing those who followed the diet with those who did not. Out of 100 patients, 26 were deceased (18 male and 8 female), while 74 were alive. The causes of death were pancytopenia and sepsis with infection and chemotherapy-induced toxicity. Out of 100 patients, the compliant group had a 27% mortality rate, compared to 23% in the non-compliant group. Of the 66% who were compliant, 18% died. The non-compliance group comprised 34% of patients, of which 8% died. Supplementary Figure 4 shows the mortality rate at a 1-year follow-up period and shows that there were no differences in mortality rates in compliant (27.3%) versus non-compliant patients (23.5%), with an overall mortality rate of 26.0%. There was a complete absence of any correlation observed between the likelihood of survival rates and the strict adherence to a neutropenic diet. This lack of correlation was also evident with equal incidence of sepsis in both groups. The underlying reasons could primarily be attributed to the presence of multiple variables and outcome measures that are associated with these patients. These patients are usually immunocompromised and hence prone to infection because of disease and chemotherapy. Furthermore, it is important to note that sepsis is not directly attributed to the dietary restrictions imposed by the neutropenic diet (9, 10).

### Conformation of diet tenacity

The total study population was divided into two groups based on adherence to dietary interventions: compliant and non-compliant (Supplementary Table 8). The compliant group consisted of 66%, strictly following a neutropenic diet, and the non-compliant group consisted of 34%. Almost everyone (99%) avoided leftover foods, 86% ate boiled egg and non-vegetarian food, 14% avoided poultry, and no patients (0%) consumed fresh fruits and raw vegetables (Supplementary Table 11). In the initial survey, 17% of the participants could not be followed up, whereas in the subsequent survey, this percentage was reduced to 10%. The majority of the parents followed neutropenic diet guidelines, but patients had difficulties with food restrictions. The observed log risk ratio ranged from −0.34 to 0.23 in the adverse medical condition population of both the compliant and non-compliant groups, while it was observed to be in the range of −0.89 to 0.08 in the non-adverse medical condition population of both the groups with the majority of estimates being positive (60%). The detailed log risk ratio and the estimated average log risk ratio, based on the random-effects model, were analyzed (Supplementary Figures 1A,B, 2A,B and Supplementary Tables 6, 7), and we observed no significant difference (*p* < 0.05) in both compliant and non-compliant groups for adverse and non-adverse medical conditions (z = 0.5359 and *p* = 0.5920 for adverse medical condition; and z = −0.1944 and *p* = 0.8458 for non-adverse medical condition) (Supplementary Tables 6, 7). According to the Q-test, there was no significant amount of heterogeneity in the true outcomes in the adverse medical condition population (Q(4) = 2.6168, *p* = 0.6239, tau^2^ = 0.0000, I^2^ = 0.0000%). A similar line of outcome was observed in the non-adverse medical condition population with the Q-test (Q(4) = 3.2983, *p* = 0.5092, tau^2^ = 0.0053, I^2^ = 8.4224%) (Supplementary Tables 6, 7). The log risk ratios and meta-analysis data are provided in Supplementary material 2 and Supplementary Tables 6, 7. The major reasons for non-compliance were logistic issues related to a large family (6%), lack of resources (4%), financial issues (4%), perceived lack of advantage (2%), not following doctors’ advice (1%), and lost to follow-up, those that were not reachable on the telephone and did not attend the follow-up (17%). Fresh fruits (excluding bananas) and raw vegetables were strictly avoided by the patients. Some parents avoided fast food, struggled to provide dry fruits due to low income, and substituted supplementary protein foods for eggs or non-vegetarian options. The majority of parents provided fresh homemade food, while a few patients consumed leftovers.

Among the 100 pediatric hematology patients studied, 66 patients were classified as compliant/adherent to dietary interventions, while 34 were non-compliant/non-adherent. The median age of compliant patients (4.32 years) was lower than that of non-compliant patients (6.67 years). Gender distribution showed that out of 61 male patients, 44 were compliant, while 17 were non-compliant. Among 39 female patients, there was an equal distribution with 22 being compliant and 17 non-compliant. Regarding disease distribution, B-ALL was the most common diagnosis (55 patients), with 35 patients in the compliant group and 20 in the non-compliant group. This was followed by T-ALL (13 patients; 10 compliant, 3 non-compliant), AML (9 patients; 6 compliant, 3 non-compliant), aplastic anemia (6 patients; 3 in each group), and Ewing sarcoma (2 patients; both compliant). The primary outcome measures of neutropenic infections during chemotherapy showed that 91 patients developed sepsis, with a higher proportion in the compliant group (62 patients) than in the non-compliant group (29 patients). Blood culture results revealed 19 positive cases (11 compliant, 8 non-compliant) and 81 negative cases (55 compliant, 26 non-compliant). The mortality data showed that out of 26 deaths, 18 were males (13 compliant, 5 non-compliant) and 8 were females (5 compliant, 3 non-compliant). The total mortality rate appeared to be similar between compliant (18 deaths) and non-compliant (8 deaths) groups when adjusted for group size. Regarding dietary interventions, nearly all patients (99 out of 100) were advised to avoid leftovers, with similar adherence rates between groups. All patients (100) were instructed to avoid fresh fruits and raw vegetables. A smaller subset of patients (14) were advised to avoid poultry (9 compliant, 5 non-compliant) and boiled eggs and non-vegetarian food (11 compliant, 4 non-compliant). The data suggest that while compliance with dietary restrictions was observed in approximately two-thirds of the patients, the incidence of neutropenic infections and positive blood cultures remained proportional between compliant and non-compliant groups when adjusted for group size. This raises interesting questions about the direct impact of dietary compliance on infection prevention in pediatric hematology and oncology patients, though other factors such as age, underlying disease, and treatment protocols may have influenced these outcomes (Supplementary Table 8).

## Discussion

The current study aimed to evaluate the impact of adherence to a neutropenic diet on morbidity, especially the frequency of hospital admissions, duration of stay. and mortality, among pediatric patients undergoing chemotherapy for ALL, AML, and other hemato-proliferative disorders. As reported in other studies, despite the theoretical benefits of reducing infection risk in patients with compromised immune function, our findings indicate that adherence to a neutropenic diet does not significantly impact the incidence of neutropenic infections or overall survival outcomes (19). There was no significant difference in morbidity and mortality between the compliant and non-compliant groups over a period of 1-year follow-up. The neutropenic diet, a norm for conventional chemotherapy for over two decades, exhibits variability in necessity, implementation, understanding, and impact on outcomes (2). The clinical significance of a neutropenic diet on pediatric hematology patients is related to the length of time needed for diet instruction, the information included in that education regarding food limitations, and the difficulty in following dietary restrictions when experiencing chemotherapeutic side effects (7).

### Patient demographics and compliance

The study cohort consisted of 100 pediatric hematology patients with a male predominance (61%), which is consistent with the existing literature (20, 21) suggesting a higher incidence of childhood cancer in males for all genetically predisposed cancers and genetic abnormalities being more common in males for unknown reasons (20, 21). It could also be potentially due to socioeconomic factors in India that influence healthcare-seeking behavior (22). The majority of patients were under the age of 10, with a peak between 0 and 5 years as expected with the published literature, with B-ALL being the prevalent diagnosis, reflecting typical disease demographics (23, 24). Fever, pallor, abdominal pain, swelling, vomiting, and diarrhea were the prevalent signs and symptoms. The male/female ratio was 1.6:1, indicating male dominance in the population. We found that compliance with the neutropenic diet was relatively high (66%), though logistical, financial, and resource-related challenges contributed to non-compliance in 34% of the cases. Interestingly, dietary compliance was notably better among children with literate parents than those with illiterate parents, emphasizing the role of parental education in health-related behavior.

In our study, 38% of mothers had completed primary education, and 24% were illiterate (Supplementary Table 4), highlighting the impact of parental education on the wellbeing of children with leukemia. Research indicates that educated parents are well-equipped to understand leukemia, chemotherapy, and its associated side effects, enabling them to provide more effective care and support (23, 25). This comprehensive understanding facilitates improved communication with healthcare providers, better management of treatment-related challenges, and reduced stress for both parents and children. Consequently, the quality of life for children is significantly enhanced when parents possess formal education, compared to relying solely on informal information sources (25).

### Diet adherence and infection outcomes

The primary outcome of the study was the incidence of neutropenic infections, defined by febrile neutropenia requiring hospital admission and broad-spectrum antibiotic treatment. The secondary outcomes included documented infections such as pneumonia and positive cultures. Although 91% of patients experienced neutropenic infections during the study, there was no clinical evidence of a major difference in infection rates between patients adherent and non-adherent to the neutropenic diet. Moreover, the types of infections, including sepsis and febrile neutropenia, were distributed randomly between both groups. In our setting, patients usually reported late, and this is a major reason for such a high incidence of sepsis-related symptoms. Sepsis was defined according to the standard definition as suspected or proven infection caused by any pathogen or clinical syndrome associated with a high probability of infection along with any two of the following four signs: fever >38.5°C, tachycardia, tachypnoea as per age-defined cutoffs, and neutropenia. Severe sepsis was defined as sepsis with organ dysfunction, hypoperfusion, or hypotension. Blood culture was positive in 19% of cases, and the details of growth are shown in the chart. One patient had polymicrobial sepsis in the form of oral candidiasis with laboratory examination showing the growth of *E. coli* and MRSA in the blood (Supplementary Figure 3).

### Morbidity, mortality, and clinical implications

In terms of clinical outcomes, the overall mortality rate was 26%, with minimal difference between the compliant (27.3%) and non-compliant (23.5%) groups (Supplementary Tables 6, 7 and Supplementary Figure 4). The primary causes of death were pancytopenia, sepsis, and chemotherapy-induced toxicity, underscoring the multifactorial nature of mortality in pediatric hematology and oncology patients. These results indicate that adherence to a neutropenic diet does not significantly reduce morbidity or mortality in pediatric hematology and oncology patients undergoing chemotherapy. Several studies including randomized controlled trials and systematic reviews have highlighted the futile effects of a neutropenic diet. However, those are mostly set in high-income countries (HIC) where the pathogen burden is less and incidences of febrile admissions due to sepsis are also low (26). In our study, about 66% followed neutropenic diet, though full compliance was not seen. The mortality rates did not vary between the compliant and non-compliant groups. In fact, the compliant group showed slightly higher mortality rates (2, 19, 27, 28). Nutrition and childhood cancer are closely linked, with malnutrition increasing the infection risk, altering medication metabolism, and limiting treatment effectiveness. A study reported that concerns about cancer outcomes, survival rates, treatment tolerance, and quality of life were identified mainly in children lacking sufficient nutrition (29). The presence of malnutrition is correlated with reduced rates of survival and heightened levels of toxicity resulting from chemotherapy treatment (23). As in any pediatric cohort in Indian tertiary care centers, we had a similar incidence of malnutrition (more than half, 54%) as per Revised IAP 2015 Growth Charts (30). The attribution of the confounding effect of malnutrition in sepsis in overall outcomes of leukemia needs a thorough investigation which is beyond the scope of this study.

### Microbiological findings and diet correlation

Blood cultures and other systemic laboratory examinations indicated that infections such as *E. coli*, MRSA, and *Klebsiella* sepsis were not specifically associated with diet adherence, highlighting that factors other than dietary intake play a predominant role in infection risk among these patients. Our findings align with other studies (15, 31) reporting high rates of culture-negative sepsis in chemotherapy patients, further suggesting that neutropenic infections are largely influenced by chemotherapy-induced immunosuppression rather than dietary factors (Supplementary Table 2). Several studies conducted in Western countries have identified common pathogens such as *Campylobacter coli*, *Bacillus cereus*, *Salmonella Typhimurium*, *Aeromonas hydrophila*, and *Staphylococcus aureus*, which were found in blood and respiratory, intestinal, and urinary sites (27, 32, 33). In addition, food-associated infections (FAIs) were notably prevalent in home environments compared to hospital settings, with studies indicating a 64% occurrence in homes versus 18% in hospitals. This variance underscores the paramount importance of domestic sanitation practices in the mitigation of foodborne illnesses (34, 35). The frequency of culture positivity in the aforementioned studies demonstrated considerable heterogeneity, ranging from 0.2 to 30%; nonetheless, it revealed analogous characteristics among both adherent and non-adherent cohorts.

The home environment serves as a crucial locus for FAIs, attributable to heterogeneous hygiene practices and the existence of varied bacterial populations. Inadequate hygienic practices, such as insufficient hand washing and improper food handling, exacerbate the elevated prevalence of FAIs in domestic environments. Conversely, healthcare facilities generally implement more rigorous hygiene protocols, thereby diminishing the occurrence of such infections. Although the incidence of FAIs is markedly elevated in domestic settings, the comparability in culture positivity rates between adherent and non-adherent groups indicates that additional factors such as environmental conditions and the adaptability of pathogens may also exert influence. This highlights the necessity for comprehensive strategies that simultaneously address individual behaviors and systemic food safety frameworks (36).

### Reasons for non-compliance

Non-compliance with the neutropenic diet was observed in 34% of the patients, primarily due to logistical challenges, financial constraints, and a lack of perceived benefits. Large family sizes made it difficult for parents to adhere strictly to dietary guidelines, especially in resource-limited settings. Financial issues also played a significant role, as some families struggled to afford the recommended foods, particularly high-protein options such as eggs or non-vegetarian items. In addition, some parents did not perceive any clear advantages to following the diet, leading to reduced motivation for strict adherence. Cultural preferences and accessibility issues further contributed to the challenges of maintaining diet compliance. Other challenges reported in the Western countries were different from those observed in our study, such as unpleasant olfactory sensations, inadequate food presentation, hospitalization, separation from family, and social pressure. Other challenges that have been reported mainly in high-income countries include complaints about dietary limits—particularly for pediatric hematology patients, who were denied desirable foods such as fast food, street foods, and fresh fruits—lengthy hospitalizations, emotional distress, disruptions to daily routines, yearning for social milieu, and dissatisfaction with meals that contribute to diminished appetite among pediatric patients (9, 16).

The study is constrained by its observational design, coupled with the relatively small sample size, factors that may significantly influence the extent to which our findings can be generalized to broader contexts beyond our study. In addition, the reliance on self-reported dietary adherence and parental recall introduces potential biases that could affect the accuracy and reliability of compliance data. The inherent limitations in our study are further compounded by factors such as a relatively brief enrollment period, generalized inclusion criteria, a limited sample size, an array of multifactorial influences, seasonal fluctuations in the availability of foodstuffs, and the notably low accuracy associated with patients’ self-reported compliance over designated intervals of 6 and 12 weeks. As a result, future research should prioritize large-scale randomized controlled trials to better establish the role and effectiveness of dietary interventions in pediatric hematology and oncology patients. In addition, it would be prudent to investigate a wider array of preventive strategies, such as the implementation of enhanced infection control mechanisms and the administration of targeted antimicrobial prophylaxis, as these may yield considerable benefits for this particularly vulnerable population of patients. There is an urgent need for further research aimed at improving the treatment outcomes associated with neutropenia, as this condition poses significant challenges in the context of oncological care. A thorough review of the existing literature indicates a pronounced deficiency in empirical evidence regarding the utilization, feasibility, and overall efficacy of the neutropenic diet specifically in pediatric patients diagnosed with neutropenia, who are actively undergoing oncological treatment.

## Conclusion

Our study data do not support the use of the neutropenic diet. Moreover, there is no standardization and recommended diet when it comes to neutropenic diet as it varies between geographic regions based on the availability of food and dietary habits. The absence of a clear link between diet and infection underscores that neutropenic infections are primarily driven by the compromised immune function inherent to chemotherapy, rather than dietary practices alone.

## Data Availability

The original contributions presented in the study are included in the article/Supplementary material, further inquiries can be directed to the corresponding authors.
