# Supplementary material for: Evaluating the efficacy and impact of neutropenic diet in pediatric hematology patients: a longitudinal cohort study on adherence, clinical outcomes, and socioeconomic factors
Source: Front Nutr. 2025 Mar 17;12:1533734. doi: 10.3389/fnut.2025.1533734 (PMC11955492; doi:10.3389/fnut.2025.1533734)
Supplement: Supplementary file 1 [file Table_1.docx]

**Supplementary Material 1 ( Figures 3,4 & Tables 1-11 )**

**Supplementary Table 1:** Pediatric hematology Patients characterized based on their gender, Age Group, ANC and Diagnosis.

| Variable | N |
| --- | --- |
| **Gender** |  |
| Females | 39 |
| Males | 61 |
| **Age groups** |  |
| 0-5 | 48 |
| 5-10 | 31 |
| 10-15 | 20 |
| **Diagnosis^1^** |  |
| B-ALL | 55 |
| T-ALL | 13 |
| AML | 9 |
| Aplastic anaemia | 6 |
| Ewing sarcoma | 2 |
| Others | 15 |
| **ANC No.^2^** |  |
| Grade1 (>1500) | 39 |
| Grade2 (1000-1500) | 3 |
| Grade3 (500-1000) | 9 |
| Grade4 (< 500) | 49 |
| **BMI Z score^3^** |  |
| Under-weight (< -1.88) | 54 |
| Normal (-1.88 to 0.54) | 33 |
| Over-weight (0.55 to < 1.34) | 1 |
| Obese (>1.34) | 4 |
| Not applicable | 8 |

Note: **^1^Diagnosis: -** B-ALL:- B-Cell Acute Lymphoid Leukaemia, T-ALL:- T-Cell Acute Lymphoid Leukaemia, AML:- Acute Myeloid Leukaemia, Others (mention in supplementary table 10) :- CML, Thalassemia, Acute ITP (Immune Thrombocytopenia), LCH (Langerhans Cell Histiocytosis), Retinoblastoma, Diffuse Leptomeningeal, BPDCN (Blastic Plasmocytoid Dendritic Cell Neoplasm), PNET (Primary Neuroectodermal Tumor), AML-NOS (Not Otherwise Specified), Lymphoma.

**^2^About Grade & Risk of Infection: -** ANC above 1500: No risk, ANC of 1000 to 1500: Minimal risk, ANC of 500 to 1000: Moderate risk, ANC less than 500: Severe/High risk

^3^**BMIZ (Body mass index for age Z score)**: IAP (Indian Academy of Pediatrics) BMI Z score is not applicable of age less than 2-year-old. The Revised IAP 2015 growth charts are suitable for Indian children due to the similarity of findings among lower socioeconomic class children to the studies

**Supplementary Table 2:** Information of Physical characteristics in pediatric patients

| **Parameter** | **N** |
| --- | --- |
| ***1. Fever*** |  |
| a)      Present | **97** |
| b)     Absent | **3** |
| ***2. Pallor*** |  |
| a)      Present | **78** |
| b)     Absent | **22** |
| ***3. Abdominal Pain/Distention*** |  |
| a)      Present | **60** |
| b)     Absent | **40** |
| ***4. Swelling^1^*** |  |
| a)      Present | **58** |
| b)     Absent | **42** |
| ***5. Bruises*** |  |
| a)     Present | **12** |
| b)     Absent | **88** |
| ***6.Vomiting*** |  |
| a)      Present | **25** |
| b)     Absent | **75** |
| ***7.Bleeding*** |  |
| a)      Present | **17** |
| b)     Absent | **83** |
| ***8. Lymphadenopathy*** |  |
| a)      Present | **64** |
| b)     Absent | **36** |
| ***9. Organomegaly^2^*** |  |
| a)      Present | **82** |
| b)     Absent | **18** |
| ***10. Evidence of Sepsis*** |  |
| a)      Present | **91** |
| b)     Absent | **9** |
| ***11. Blood Culture^3^*** |  |
| a)      Positive | **19** |
| b)     Negative | **81** |
| ***12. Others^4^*** |  |
| a)      Present | **77** |
| b)     Absent | **23** |

Note:^1^Swelling (present in any part of the body)

^2^Organomegaly include Hepatosplenomegaly (63%), Hepatomegaly (16%), Splenomegaly (2%) and Hydronephrosis (1%).

^3^Infection presents in blood culture include Klebsiella, Candida, MRSA, Enterococcus, Acinetobacter, Staphylococcus, and E. coli. When blood is cultured, bacteria from the bloodstream are represented as positive results. One Patient had Oral Candidiasis with E. coli Sepsis & MRSA sepsis. Another one had Acinetobacter Sepsis. Some Patient had Febrile neutropenia with Klebsiella Sepsis.

^4^Others parameters include like Cold, Cough, running nose, Loose stool, Difficulty in walking, Headache, Body ache, Blurry vision, redness in eye, Throat pain, Ear discharge, Appetite loss, weakness, Nausea, Taste alteration, Difficulty in breathing.

**Supplementary Table 3.** Profile and outcomes among various types of leukaemia in our study

| **Diagnosis** | **Male/ Female Ratio** | **Number of**  **Patients** | **Underweight % based on BMI Z Score** | **Compliance Percentage**  **%** | **Admission with Sepsis %** | **Blood Culture %** | **Death %** | **Alive %** |
| --- | --- | --- | --- | --- | --- | --- | --- | --- |
| **B-ALL** | 1.5:1 | 55 | 50.9 | 63.6 | 23.6 | 23.6 | 27.3 | 72.7 |
| **T-ALL** | 3:1 | 13 | 61.5 | 76.9 | 23.1 | 07.7 | 0 | 100 |
| **AML** | 1:1 | 9 | 55.5 | 66.7 | 22.2 | 22.2 | 11.2 | 88.8 |
| **Aplastic anaemia** | 5:1 | 6 | 66.7 | 50 | 16.7 | 16.7 | 33.4 | 66.6 |
| **Ewing Sarcoma** | 1:2 | 2 | 100 | 100 | 0 | 0 | 50 | 50 |
| **Others** | 1:1 | 15 | 46.7 | 66.7 | 0 | 13.33 | 47.7 | 53.3 |

**Supplementary Table 4:** Baseline demographic data of patient’s parents included in the study

|  | **N** |
| --- | --- |
| ***Age of parents (years)*** |  |
| Between 20-30 | **74** |
| Between 30-40 | **24** |
| Between 40-50 | **2** |
| ***Mother Literacy*** |  |
| Illiterate | **24** |
| Primary Passed | **38** |
| Secondary Passed | **27** |
| Graduate | **11** |
| ***Father Literacy*** |  |
| Illiterate | **32** |
| Primary Passed | **8** |
| Secondary Passed | **44** |
| Graduate | **16** |
| ***Occupation of mother*** |  |
| Household/Not working | **92** |
| Skilled worker | **3** |
| Self Employed | **1** |
| Labour | **4** |
| ***Occupation of father*** |  |
| Household | **3** |
| Skilled worker | **53** |
| Self Employed | **28** |
| Labour | **16** |
| ***Profession of attending health care staff*** |  |
| Doctors (3Jr, 3 Sr.) | **6** |
| Nurses | **6** |
| Support staffs | **2** |

**Supplementary Table 5**. Details of questionnaire at the time of starting of neutropenic diet and follow-

| **QUESTIONS** | **YES** | **NO** |
| --- | --- | --- |
| 1. Have you ever consumed food in a neutropenic manner? 2. Do you ever check expiry date of Packed food? 3. Do you have refrigerator or have you ever checked your refrigerator's temperature? 4. Do you use leftover food on next day? 5. Do you check damaged packaging of meat & fish and do you keep fish, meat or egg in refrigerator? 6. Do you wash your hand with soap before & after handling or eating or cooking food? 7. Do you clean work surface daily? 8. Do you rinse all vegetables with clean running water prior to use? 9. Do you eat raw vegetables, salads, raw fruits, or outside vendors food & juices? 10. Do you eat nuts & seeds or dried fruit? 11. Do you drink milk? 12. Do you eat Meat & Poultry (Mutton, Chicken)? 13. Do you eat outside packed food (Noodles, macaroni, chips, Pasta)? 14. Do you consume Egg(boiled), Fish and seafood (All types of fish, prawns, crabs etc)? 15. Do you consumed foods made from grains? (rice, chapati, bread etc) 16. Do you consume white roots and tubers and plantains (Potatoes, beet root, radish)? 17. Do you eat Pulses? (All types of Dals) 18. Do you consume packed fruit juices and milk products? (paneer, curd) 19. What did you eat for breakfast, lunch and dinner? |  |  |

**Supplementary Table 6:** Meta-analysis of the incidence of adverse medical condition

| **Medical Condition** | **STUDY GROUP** | **CONTROL** | | | **Risk Ratio, 95% CI** |
| --- | --- | --- | --- | --- | --- |
|  | **EVENT** | **TOTAL** | **EVENT** | **TOTAL** |  |
| Neutropenic infections during chemotherapy | 62 | 66 | 29 | 34 | 0.10 [-0.06, 0.25] |
| Fever | 64 | 66 | 33 | 34 | -0.00 [-0.07, 0.07] |
| Diarrhoea | 27 | 66 | 11 | 34 | 0.23 [-0.33, 0.80] |
| Other symptoms | 51 | 66 | 26 | 34 | 0.01 [-0.22, 0.24] |
| Culture positive infections | 11 | 66 | 8 | 34 | -0.34 [-1.16, 0.47] |
|  |  |  |  |  |  |
| Total 95% CI |  | | | | 0.0170 [-0.0453, 0.0793] |
| Heterogeneity | (Q (4) = 2.6168, p = 0.6239, tau² = 0.0000, I² = 0.0000%). | | | | |
| Test for overall effects | z = 0.5359, p = 0.5920 | | | | |

**Supplementary Table 7:** Meta-analysis of the incidence of no adverse medical condition

| **Medical Condition** | **STUDY GROUP** | **CONTROL** | | | **Risk Ratio, 95% CI** |
| --- | --- | --- | --- | --- | --- |
|  | **EVENT** | **TOTAL** | **EVENT** | **TOTAL** |  |
| Neutropenic infections during chemotherapy | 4 | 66 | 05 | 34 | -0.89 [-2.13, 0.36] |
| Fever | 2 | 66 | 01 | 34 | 0.03 [-2.33, 2.39] |
| Diarrhoea | 39 | 66 | 23 | 34 | -0.14 [-0.44, 0.17] |
| Other symptoms | 15 | 66 | 08 | 34 | -0.03 [-0.79, 0.72] |
| Culture positive infections | 55 | 66 | 26 | 34 | 0.09 [-0.13, 0.30] |
|  |  |  |  |  |  |
| Total 95% CI |  | | | | -0.0194 [-0.2147, 0.1760] |
| Heterogeneity | Q (4) = 3.2983, p = 0.5092, tau² = 0.0053, I² = 8.4224%) | | | | |
| Test for overall effects | z = -0.1944, p = 0.8458) | | | | |

**Supplementary table 8.** Based on adherence to the dietary intervention the total study population was divided into two groups- Compliant & Non-Compliant. The details of characteristics of these groups are mentioned in here

| **S.no** | **Parameters** | **Pediatric hematology patients**  **(n=100)** | **Compliant**  **or**  **Adherent group (n=66)** | **Non-compliant or**  **Non-adherent group (n=34)** |
| --- | --- | --- | --- | --- |
| **1.** | **Age** | Age (in median) | 4.32 | 6.67 |
| **2.** | **Gender** | Male (61) | 44 | 17 |
|  |  | Female (39) | 22 | 17 |
| **3.** | **Diagnosis** | B-ALL (55) | 35 | 20 |
|  |  | T-ALL (13) | 10 | 3 |
|  |  | AML (9) | 6 | 3 |
|  |  | Aplastic Anaemia (6) | 3 | 3 |
|  |  | Ewing sarcoma (2) | 2 | 0 |
|  |  | Others (15) | 10 | 5 |
| **4.** | **Neutropenic infections during chemotherapy** | Sepsis (91) | 62 | 29 |
| **5.** | **Blood culture** | Positive (19) | 11 | 8 |
|  |  | Negative (81) | 55 | 26 |
| **6.** | **Death** | Male (18) | 13 | 5 |
|  |  | Female (8) | 5 | 3 |
| **7.** | **Dietary intervention** | Avoid Leftover (99) | 66 | 33 |
|  |  | Avoid poultry (14) | 9 | 5 |
|  |  | Avoid Fresh fruits (100) | 66 | 34 |
|  |  | Avoid Raw vegetables (100) | 66 | 34 |
|  |  | Avoid Boiled egg & non-veg (14) | 10 | 4 |

**Supplementary table 9.** Shows the guidelines to prevent food born illness and our question in survey based on these guidelines regarding to their neutropenic diet and Hygiene.

| **S.no.** | **Parameters** | **Guidelines** |
| --- | --- | --- |
| **1.** | **Safe Shopping** | - To check date of expiry of packed food. If the "sell by" or "best used by" date has gone, do not purchase the item. - Never select anything from shredded or leaky packaging, dented cans, or shattered jars. - Use of food grade plastic bags for Packaging of raw meat, Fish, Sea foods & Poultry so that fluids won’t contaminate the food items. Do not buy meat, Sea foods & Fish in damaged packing. |
| **2.** | **Safe storing** | - Have a refrigerator and maintain temperature. - Keep fish, Sea food, meat or egg in refrigerator. To prevent fluids from dripping onto other items, put raw fish, poultry and meat in the bottom of the refrigerator. - Use leftover food next day. |
| **3.** | **Safe preparation** | - Wash your hands with soap before/ after cooking - Wash your hands with soap before/ after eating - Clean your work surface daily - Always rinse fruits and vegetables with clean running water before consuming |
| **4.** | **Avoid following Foods** | - Raw Vegetables: - All uncooked vegetables and salads should be avoided. Even foods like Maggie, pasta and sandwiches should be free from raw vegetables. - Raw Fruits: - Avoid raw fruits and consume only fresh fruits with thick covering like banana and orange. - Avoid fruit juices and raw milk – Don’t consume any fruit juices and only boiled milk should be consumed. - Outside foods like street food including foods from restaurants should be avoided - Cooked & Prepared Fast Foods should be avoided as much as possible - Raw or semi-cooked Fish, Sea foods & Meat should be avoided |
| **5.** | **Diet of Day** | - Ask what you have in Breakfast, what will you have in Lunch, Evening Breakfast & Dinner |

**Supplementary table 10.** List of the number of patients with diagnosis using the term "others”

| S.no | Diagnosis (Others) | N=15 |
| --- | --- | --- |
| 1 | Acute ITP (ImmuneThrombocytopenia) | 1 |
| 2 | AML-NOS (Not Otherwise Specified) | 1 |
| 3 | CML (Chronic Myeloid Leukemia) | 3 |
| 4 | Diffuse Leptomeningeal | 1 |
| 5 | LCH (Langerhans Cell Histiocytosis) | 2 |
| 6 | Lymphoma | 2 |
| 7 | Retinoblastoma | 1 |
| 8 | Thalassemia | 2 |
| 9 | PNET (Primary Neuroectodermal Tumor) | 1 |
| 10 | BPDCN (Blastic Plasmocytoid Dendritic Cell Neoplasm) | 1 |

**Supplementary table 11.** Neutropenic diet parameters with compliance and non-compliance rate

| S.No. | **Some parameters of Neutropenic diet** | **No. of patients who avoid**  **(n=100)** | **No. of patients who consume**  **(n=100)** |
| --- | --- | --- | --- |
| 1 | Leftover food | 99 | 1 |
| 2 | Poultry related food | 14 | 86 |
| 3 | Boiled egg & non-veg food | 14 | 86 |
| 4 | Fresh fruits | 100 | 0 |
| 5 | Raw vegetables | 100 | 0 |

**Supplementary figure 3:** In our setting patients usually report late and this is a major reason for such high incidence of sepsis related symptoms. Sepsis was defined as per standard definition as – suspected or proven infection caused by any pathogen or clinical syndrome associated with high probability of infection along with any two of following 4 signs - Fever >38.5 C, tachycardia, tachypnoea as per age defined cut offs and neutropenia. Severe sepsis was defined as sepsis with organ dysfunction, hypoperfusion or hypotension.

Blood culture was positive in 19% cases and the details of growth have been shown in the chart. One patient had polymicrobial sepsis in form of oral Candidiasis with blood showing growth of E. coli & MRSA.

**Supplementary figure 4:** Details of dead and alive patients with regard to compliance and non-compliance.
