# Supplementary material for: Evaluating the efficacy and impact of neutropenic diet in pediatric hematology patients: a longitudinal cohort study on adherence, clinical outcomes, and socioeconomic factors
Source: Front Nutr. 2025 Mar 17;12:1533734. doi: 10.3389/fnut.2025.1533734 (PMC11955492; doi:10.3389/fnut.2025.1533734)
Supplement: Supplementary file 2 [file Table_2.docx]

**Supplementary Material 2 (Statistical analysis results and Figure 1& 2)**

**Statistical analysis part of the study**

**Methodology:**

The statistical analysis of the study was conducted using Jamovi-open statistics software (Version: 2.3.28, Solid). A p value less than 0.05 was the threshold for statistical significance. The study was conducted with the logarithmic risk ratio as the measure of final outcome. The data was fitted with a random-effects statistical model. We evaluated the level of heterogeneity (tau²) using the constrained maximum-likelihood estimator. Additional to the tau² estimate, the Q-test for heterogeneity and the I² statistic are also provided. If any degree of heterogeneity is seen (i.e., tau² > 0 irrespective of the Q-test findings), a prediction interval for the actual effects is also given. The utilization of studentized residuals and Cook's distances allows for the examination of potential outliers and/or influential studies within the model's context. Studies exhibiting a studentized residual in excess of the 100 x (1 - 0.05/(2 X k))th percentile of a regular normal distribution are regarded as possible outliers. This is determined by applying a Bonferroni correction with a two-sided alpha of 0.05 to the k studies included in the meta-analysis. Influential studies are defined as those with a Cook's distance greater than the median plus six times the interquartile range of the Cook's distances. We employ the rank correlation test and the regression test, with the standard error of the observed results as the predictor, to assess funnel plot asymmetry (Ma et al., 2022; Viechtbauer, 2010).

**Results:**

A total of k=5 studies (5 different medical conditions) were included in the analysis for both adverse medical condition and No-adverse medical condition population for both neutropenic diet and standard diet population. The observed log risk ratios ranged from -0.3448 to 0.2346, in adverse medical condition population in both the experimental and control group while it was observed from -0.8864 to 0.0859 in No-adverse medical condition in both the experimental and control group with the majority of estimates being positive (60%). The estimated average log risk ratio based on the random-effects model was \hat{\mu} = 0.0170 (95% CI: -0.0453 to 0.0793) (**Table 6; Fig. 1A**) for adverse medical condition population and -0.0194 (95% CI: -0.2147 to 0.1760) (**Table 7; Fig. 2A**) for No-adverse medical condition population. So, the average outcome did not differ significantly from zero in both adverse medical condition and No-adverse medical condition population (z = 0.5359, p = 0.5920; for adverse medical condition) and (z = -0.1944, p = 0.8458; for No-adverse medical condition) (**Table 6 & Table 7**). According to the Q-test, there was no significant amount of heterogeneity in the true outcomes in the adverse medical condition population (Q(4) = 2.6168, p = 0.6239, tau² = 0.0000, I² = 0.0000%) (**Table 6**). One study (Fever) had a relatively large weight compared to the rest of the studies (i.e., \mbox{weight} \ge 3/k, so a weight at least 3 times as large as having equal weights across studies) (**Fig. 1A**). An examination of the studentized residuals revealed that none of the studies had a value larger than ± 2.5758 and hence there was no indication of outliers in the context of this model (**Fig. 1A**). According to the Cook's distances, one study (Fever) could be considered to be overly influential. Neither the rank correlation nor the regression test indicated any funnel plot asymmetry (p = 1.0000 and p = 0.8004, respectively) (**Fig.1B**). Similar line of outcome was appeared in the No-adverse medical condition population with the Q-test, with no significant amount of heterogeneity in the true outcomes (Q(4) = 3.2983, p = 0.5092, tau² = 0.0053, I² = 8.4224%) (**Table 7**). A 95% prediction interval for the true outcomes is given by -0.2614 to 0.2226. Hence, although the average outcome is estimated to be negative, in some studies the true outcome may in fact be positive. An examination of the studentized residuals revealed that none of the studies had a value larger than ± 2.5758 and hence there was no indication of outliers in the context of this model. Here also according to the Cook's distances, none of the studies could be considered to be overly influential. Neither the rank correlation nor the regression test indicated any funnel plot asymmetry (p = 0.8167 and p = 0.2982, respectively) (**Fig. 2B**).

**Reference:**

Ma, Y., Lu, X., Liu, H., 2022. Neutropenic Diet Cannot Reduce the Risk of Infection and Mortality in Oncology Patients With Neutropenia. Front. Oncol. 12, 836371.

Viechtbauer, W., 2010. Conducting meta-analyses in R with the metafor package. J. Stat. Softw. 36(3), 1-48.


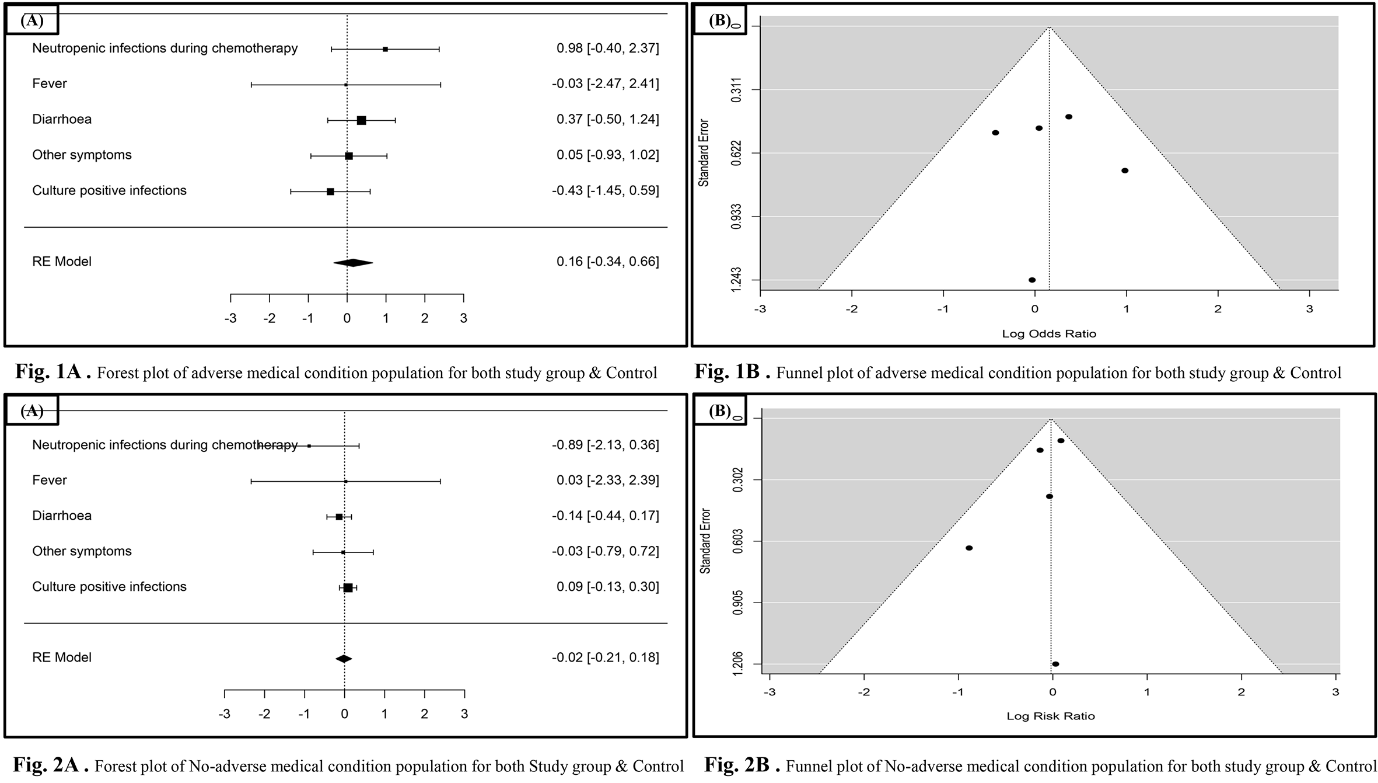


**Supplementary figure 1 & 2:** Forest & funnel plot of adverse and non-adverse medical conditions
